# Primary Prevention of Type 2 Diabetes Mellitus in the European Union: A Systematic Review of Interventional Studies

**DOI:** 10.3390/nu17061053

**Published:** 2025-03-17

**Authors:** Carlos Alexandre Soares Andrade, Szabolcs Lovas, Nour Mahrouseh, Ghenwa Chamouni, Balqees Shahin, Eltayeb Omaima Awad Mustafa, Abdu Nafan Aisul Muhlis, Diana Wangeshi Njuguna, Frederico Epalanga Albano Israel, Nasser Gammoh, Niyati Chandrika, Nkunzi Conetta Atuhaire, Israa Ashkar, Anoushka Chatterjee, Rita Charles, Hasan Alzuhaily, Alaa Almusfy, Daniela Díaz Benavides, F. K. Alshakhshir, Orsolya Varga

**Affiliations:** 1 Department of Public Health and Epidemiology, Faculty of Medicine, University of Debrecen, 4028 Debrecen, Hungary; soares.andrade@med.unideb.hu (C.A.S.A.); lovas.szabolcs@med.unideb.hu (S.L.); ghenwa.chamouni@med.unideb.hu (G.C.); balqeesalturk.7.11@hotmail.com (B.S.); omaima.awad@med.unideb.hu (E.O.A.M.); nafan.abdu@med.unideb.hu (A.N.A.M.); frederico.israel@med.unideb.hu (F.E.A.I.); nassergammoh@googlemail.com (N.G.); niyatichandrika20@gmail.com (N.C.); nkonetta@gmail.com (N.C.A.); anoushkachatt@gmail.com (A.C.); charles1914@mailbox.unideb.hu (R.C.); hasanalsohaily@gmail.com (H.A.); alaaalmosfi@gmail.com (A.A.); diazdaniela@mailbox.unideb.hu (D.D.B.);; 2 Doctoral School of Health Sciences, University of Debrecen, 4032 Debrecen, Hungary; 3 Medical Surgical Department, School of Nursing, Dedan Kimathi University of Technology, Nyeri 10143, Kenya; diana.njuguna@med.unideb.hu; 4 Department of Stomatology, Faculty of Medicine and Dentistry, Universitat de València, 46010 Valencia, Spain; isash@alumni.uv.es

**Keywords:** type 2 diabetes mellitus, systematic review, European Union, incidence, primary prevention, interventional studies

## Abstract

Interventions for primary prevention are crucial in tackling type 2 diabetes (T2D) by offering a structured approach to implementing lifestyle modifications, such as community-based programs. The aim of this study was to demonstrate the effectiveness of primary prevention interventions in preventing or delaying the onset of T2D in the 28 EU member states (EU-28). The present systematic review is registered on PROSPERO (CRD42020219994), and it followed the PRISMA guidelines. Eligibility criteria comprised original interventional studies reporting incidence of T2D in member states of the EU-28. A total of 23,437 records were initially retrieved, of which 16 met the eligibility criteria for inclusion. These interventional studies, published between 2003 and 2021, provided data from Spain, the UK, Finland, the Netherlands, and Denmark. Thirteen studies were of low quality, two were moderate, and one was high-quality. Three studies focused solely on dietary interventions, twelve studies combined diet, physical activity, and lifestyle counseling, and one study applied repeated health checks with personalized feedback and lifestyle advice. Overall, 10 studies reported a significant reduction in T2D incidence exclusively among high-risk individuals following the interventions with HR: 0.4 (95% CI: 0.3–0.7) to 0.75 (95% CI: 0.58–0.96). Only a few studies reported that primary lifestyle interventions decreased T2D risk, thus limiting generalizability. While lifestyle improvements were noted on high-risk groups, significant risk reduction among healthy individuals was not observed. Multicomponent interventions combining dietary modifications, physical activity, and personalized lifestyle counseling were the most effective in reducing the incidence of T2D among high-risk populations in the EU-28.

## 1. Introduction

According to the Global Burden of Disease 2021 study, type 2 diabetes mellitus (T2D) was responsible for over 104.000 deaths and 4.3 million Disability-Adjusted Life Years (DALYs) in the European Union (EU) [1]. In 2021, the total health expenditure of diabetes (DM) in adults (20–79 years) in the European region was estimated at USD 189.3 billion, which comprises 19.6% of global expenditure [2]. The cost of DM in the EU alone was estimated at EUR 150 billion in 2019 [3]. This includes both direct costs, such as medication and treatment, and indirect costs, such as productivity loss resulting from absenteeism and premature retirement, as well as the costs of rehabilitation and retraining. These costs exceed 10% of healthcare budgets in some EU countries [4].

The prevention of T2D is largely achievable through the implementation of lifestyle modifications, including the maintenance of a healthy diet, regular physical activity, and weight management. Studies have demonstrated that the adoption of a healthy lifestyle can significantly reduce the risk of developing T2D [5]. It is important to note that the incidence of T2D is associated with a number of risk factors, including modifiable and non-modifiable factors. Non-modifiable factors include sex, age, ethnicity, and family history. Modifiable factors include lifestyle-related factors such as cigarette smoking and an unhealthy diet, as well as physical inactivity. Obesity, in particular, is considered to be a significant metabolic risk factor for T2D [6].

The field of primary prevention of T2D presents a significant challenge from a policy perspective. One of the key obstacles is the implementation of lifestyle interventions in practice. Individuals from lower socioeconomic backgrounds are more likely to engage in negative lifestyle habits, such as smoking, physical inactivity, obesity, and low fruit and vegetable consumption. Furthermore, there is a risk that policies may fail to adequately consider the complex sociocultural environment that affects health and illness. This may lead to an overemphasis on the individual’s responsibility for lifestyle change [7]. Furthermore, it is crucial to develop behavior change and maintenance strategies that are tailored to individuals with prediabetes within their sociocultural environments. This is of paramount importance both for individuals and society as a whole in order to reduce the risk of progression to T2D. Furthermore, policy makers should propose incentives to influence health behavior among the population and those at high risk of T2D. These may include legislation, information campaigns, and price signals [8,9].

Beyond the member states, the EU has the potential to improve the health of the EU population. Notwithstanding the significant prevalence of T2D and its substantial economic impact, the EU has devoted limited policy attention to the disease. A legal surveillance study identified 22 legislations in the EU aimed at preventing DM, non-communicable diseases (NCDs), and obesity. However, only five of these legislations specifically addressed preventing DM [10]. The European Parliament passed a resolution emphasizing the prevention, management, and better care of DM in the EU. This resolution highlights the need for increased efforts to address the growing burden of DM and improve care for individuals living with the condition [11]. However, it should be noted that a number of initiatives exist that are focused on combating NCDs, covering DM, in the EU, including the Healthier Together EU NCD Initiative and the EU4health program. These initiatives provide funding for research and development of projects designed to combat NCDs [12].

Interventional studies are of significant importance in the field of DM prevention research, as they offer a structured approach to implementing specific interventions, such as lifestyle modifications and community-based programs. By directly assessing the effectiveness of such interventions, they provide crucial evidence on the value of prevention of DM [13,14]. The discussion on T2D dates back a century, yet rigorous randomized clinical trials (RCTs) for prevention only emerged in the 1990s, with earlier attempts lacking experimental designs. Limited by the absence of consensus on definitions, progress in population studies and RCTs was hindered, leading to a reliance on ecological and observational data to understand the impact of lifestyle factors on DM development [15].

The Finnish Diabetes Prevention Study (DPS) marked a significant milestone as the initial well-designed RCT focusing on preventing T2D through lifestyle changes with individual randomization. Remarkably, participants who diligently followed the lifestyle intervention and achieved four or five out of the five lifestyle goals remained free from developing T2D throughout the trial [15]. While there is substantial evidence demonstrating the efficacy of DM prevention programs, there are significant knowledge gaps pertaining to their implementation, transferability, and scalability in real-world settings. This is particularly evident in the context of addressing disparities in DM prevention and care [5].

A systematic review of interventional studies in the EU is crucial to consolidate and analyze evidence-based interventions for preventing T2D in the EU population, providing valuable insights into the effectiveness of various preventive strategies. This can guide policy-making decisions and contribute to the development of targeted and evidence-based DM prevention programs, tailored to the specific needs of the EU population. The aim of this systematic review is to identify and evaluate interventions designed to prevent or delay T2D among healthy and high-risk individuals, implemented and evaluated in the EU-28.

## 2. Materials and Methods

On 1 January 2023, the systematic review was initiated after submitting and receiving approval for the protocol on the International Prospective Register of Systematic Reviews (PROSPERO) under the identifier CRD42020219994. In order to guide each step of the methodological process and reporting, we used the Preferred Reporting Items for Systematic Reviews and Meta-Analyses (PRISMA) 2020 checklist.

### 2.1. PICOs and Research Question

Since we aimed to analyze intervention data related to the prevention of T2D, we utilized the PICOS framework (P: population; I: intervention; C: comparator; O: outcomes; S: type of study) to structure our analysis.

- Population: individuals of all age groups without DM living in the 28 member states of the EU.
- Intervention: we aimed to analyze a wide range of interventions related to physical activity regimen, dietary modifications, weight management programs, eating patterns, counseling sessions, change in attitudes, knowledge gain, supplement intake, glycemia monitoring protocols, and other preventive measures to reduce the incidence of T2D.
- Control: each intervention was compared with a control group, which could be the absence of the intervention, conventional treatments, or varying levels of the same intervention.
- Outcomes: the primary outcome measure was the incidence of T2D.
- Types of study: only interventional studies were considered for inclusion in our analysis, including but not limited to randomized clinical trials (RCTs), non-randomized trials, quasi-experimental studies, and clinical trials.

Utilizing the PICOS framework as a basis, the research questions for this systematic review were formulated:

1. Among individuals without DM residing in the EU-28, what is the effectiveness of preventive interventions compared to standard care in reducing the incidence of T2D?
2. How do different preventive measures targeting nondiabetic populations in the EU-28, such as weight loss programs, dietary supplements, counseling, physical activity, or exercise regimens, compare in terms of their ability to lower the incidence of T2D?

### 2.2. Eligibility Criteria

The eligibility assessment for titles, abstracts, and full texts adhered to a specific sequence of inclusion criteria. To be included in the present systematic review, studies should meet the following inclusion criteria: (a) original research with available full text; (b) studies with individuals living in the EU-27 member states or the UK (EU-28); (c) interventional studies, such as RCTs and non-randomized clinical trials; (d) minimum follow-up of 6 months; (e) studies assessing data on individuals without DM at baseline; (f) studies reporting T2D preventive interventions such as dietary modifications, physical activity regimens, counseling sessions, weight management programs, eating pattern, change in attitudes, knowledge gain, supplement intake, glycemia monitoring protocols, and others; and (g) studies reporting data on the onset of T2D, such as incidence rate, hazard ratio, odds ratio, and relative risk ratio.

Studies that met the following exclusion criteria were excluded: (a) observational studies such as cohort, case–control, cross sectional, economic studies, nonexperimental studies, studies of simulation models, case reports, series of cases, literature reviews, nonoriginal studies, laboratorial studies, expert opinions, and letters; (b) studies merging data from different member states; (c) studies in which the data regarding T2D were merged with different types of DM or studies in which the type of DM was not explicitly reported; (d) studies primarily focusing on any topic other than T2D; (e) studies with no relevant intervention, such as pharmaceutical, genetics, biomarkers, and risk factors; and (f) studies with lack of 95% confidence intervals (95% CIs), standard deviation (SD), or standard error (SE).

### 2.3. Search Strategy, Study Selection, and Data Extraction

With a focus on the EU-28, the search strategy for the study was centered around three main keyword concepts: T2D prevention, prevention activities (such as dietary changes, physical activities, counseling, and others), and study design (such as interventional and clinical trials). The authors created comprehensive search strategies using a combination of subject headings and keywords customized for each respective database: PubMed via EMBASE, Scopus, CINAHL Plus, Web of Science, CAB abstracts, and Clinicaltrials.gov. To validate the search strategy, the authors compared it against a set of known included articles derived from previously published reviews. Also, the list of references from the identified relevant articles was screened. A supplementary snowball search involved screening the references of similar systematic reviews. The search started on 1st January and concluded on 1 March 2023, and the retrieved results were exported in RIS format and imported into the Covidence platform (accessible at www.covidence.org) to remove duplicate articles. Articles presenting redundant information in comparison to previously included articles were systematically excluded as duplicates. This criterion was also applied to pre-print articles that had undergone peer review and publication. In such cases, preference was given to the peer-reviewed versions as the definitive source. The search strategy was based on the PICOS frameworks, and the most appropriate MeSH terms and Boolean operators were used:

“(((observational study) OR (clinical trial) OR (clinicaltrial) OR (“Clinical Studies as Topic”) OR (Controlled Before-After Studies) OR (Case-Control Studies) OR (“Cohort Studies”)) AND (type 2 diabetes mellitus)) AND ((“diet, food, and nutrition” OR “Risk Factors”) OR “Life Style” OR “Exercise” OR “Leisure Activities” OR “Preventive Health Services” OR “Communication” OR “Health Behavior” OR “Risk Reduction Behavior” OR “Dietary Supplements” OR “primary prevention” OR “prevention and control” OR “Social Determinants of Health”)) AND (Austria OR Belgium OR Bulgaria OR Croatia OR Cyprus OR Czech Republic OR Denmark OR Estonia OR Finland OR France OR Germany OR Greece OR Hungary OR Ireland OR Italy OR Latvia OR Lithuania OR Luxembourg OR Malta OR Netherlands OR Poland OR Portugal OR Romania OR Slovakia OR Slovenia OR Spain OR Sweden OR “United Kingdom” OR Europe OR “European Union”))”.

Supplementary File S1 presents the customized search strategies utilized for every electronic database, alongside the respective count of studies retrieved from each database.

Twenty trained reviewers collaborated in pairs, and three reviewers mediated disagreements in meetings for the research selection process. The process began with a pilot screening of thirty studies, which allowed reviewers to get calibrated as they evaluated the same publications together. The interrater reliability was then assessed using Cohen’s kappa coefficient, and meetings were held to resolve conflicts and standardize the screening process. After calibration, each of the 20 reviewers started using the Covidence platform to select papers, first by title and abstract. All the disagreements were solved in a consensus meeting with the two reviewers involved in the disagreement and a third reviewer with more experience on the topic. A specific order of exclusion criterion was applied for the screening process in Covidence: (a) full text is not available, (b) irrelevant article type, (c) not in the EU-28, (d) irrelevant design, (e) irrelevant topic, (f) irrelevant intervention, and (e) irrelevant outcome. While screening titles and abstracts, reviewers classified the results as “no”, “maybe”, or “yes”. When “no” was selected, the screening process for that study ended. For the other two possibilities, further screening occurred. After all abstracts were screened, the full text of the studies included was retrieved, downloaded, and uploaded to Covidence. The full-text screening phase in the systematic review was conducted using the same methodology as the title and abstract screening. Reviewers were calibrated using 10 papers, and a meeting was held to standardize the screening process. The eligibility criteria were maintained in the same order, but the “maybe” option was not available. To facilitate data extraction, one of the authors created a standardized data extraction form on Covidence based on the JBI Manual for Evidence Synthesis, which reviewers completed in pairs. The form included questions related to study characteristics (e.g., first author, publication year, and country of origin, database or data source, ethics approval), study methods (e.g., aims of the study, country, setting, study design, intervention, and follow-up), and sample characteristics (e.g., size, groups, age range, gender, diagnostic methods, and/or definitions of T2D).

### 2.4. Methodological Quality Assessment and Data Analysis

The assessment of methodological quality took place during the course of the data extraction process. Reviewers were able to extract data and complete the quality assessment form simultaneously in Covidence. We employed the National Institutes of Health’s (NIH) National Heart, Lung, and Blood Institute (NHLBI) quality assessment tool. In 2013, NHLBI developed a series of specific assessment tools that assist reviewers in focusing on concepts that are critical to a study’s internal validity. The tools were assessed to identify methodological or implementation problems, and they were adjusted according to specific study designs. The studies included in our selection were assessed using the “Quality Assessment of Controlled Intervention Studies”, which included 14 questions [16].

Reviewers completed the forms in pairs, and disagreements were settled in meetings with a third reviewer. The meeting included a consensus for the data extraction form and the quality assessment form at the same time. The authors identified a set of highly critical domains for promoting more accurate quality assessments of the included studies: items 2, 6, 9, and 11.

- Low Methodological Quality:
  ◦ Critical domain questions: One or more “no” answers, or one “no” answer combined with one “cannot determine/not reported”, or two or more “cannot determine/not reported” answers.
  ◦ Non-critical domain questions: Three or more “no” answers, or four or more “cannot determine/not reported”, or one “cannot determine/not reported” response combined with two “no” answers.
- Moderate Methodological Quality:
  ◦ Critical domain questions: One “no” answer and one “cannot determine/not reported” answer.
  ◦ Non-critical domain questions: Two “no” answers, or three “cannot determine/not reported”, or one “cannot determine/not reported” response combined with one “no” answer.
- High Methodological Quality:
  ◦ Critical domain questions: No “no” answers and no “cannot determine/not reported”.
  ◦ Non-critical domain questions: One “no” answer and at most two “cannot determine/not reported” responses.

The interrater reliability rate for each phase of the study was automatically calculated with Cohen’s kappa coefficient (κ) by the Covidence platform.

## 3. Results

**Figure 1:**
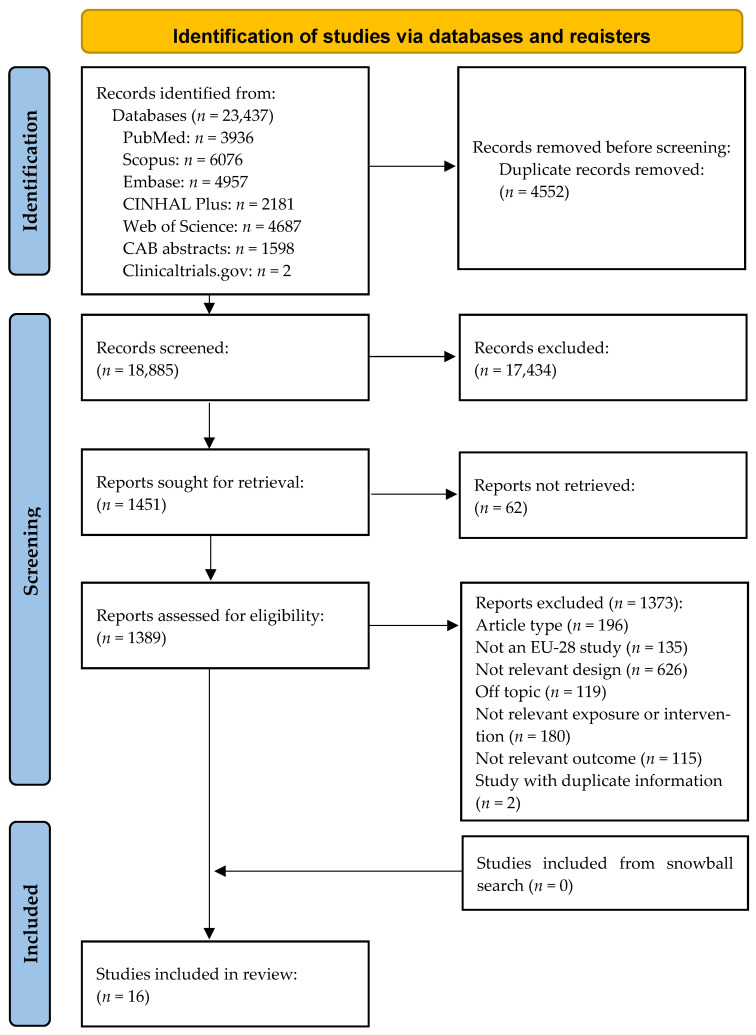
Preferred reporting items for systematic reviews and meta-analyses (PRISMA 2020).

The process of the literature search is presented in a PRISMA flow diagram (Figure 1). Our initial database search yielded 23,437 records, of which 4552 were found to be duplicates and therefore excluded. No additional records were identified by the snowball search method. Independent reviewer pairs screened 18,885 titles and abstracts, from which 17,434 were excluded, as they were found to be irrelevant to the study’s objectives. Full text was not available for 62 studies. A total of 1389 full texts were matched to the inclusion criteria and analyzed by the reviewer pairs. Out of these, 1373 did not meet the eligibility criteria, resulting in the selection of 16 studies for inclusion in our systematic review.

### Characteristics of the Included Studies

The 16 interventional studies included in our review were published between 2003 and 2021 (Table 1).

The studies included data from five distinct nations, with the distribution of papers as follows: Spain—5 [21,22,23,24,25], UK—5 [26,27,28,29,30], Finland—3 [18,19,20], The Netherlands—2 [31,32], and Denmark—1 [17]. The studies included in this analysis were subjected to reliability and quality assessment, and the results are presented in Table 2. Out of the 16 studies, 13 were found to be of low quality [17,21,22,25,26,27,28], 2 were moderate [24,26,27] and 1 was of high quality [23]. The Cohen’s kappa coefficient (κ) was 0.62 for the interrater reliability assessment, indicating moderate level of internal consistency [33]. Additional results, including ethical approval, T2D diagnosis, associated comorbidity, and other characteristics, can be found in Table 3.

The detailed primary findings from the included interventional studies are summarized in Table 4. Out of the 16 studies, 3 aimed solely at changing dietary habits without interventions targeting physical activity or caloric restriction, including individuals at high risk for T2D [21,23,25]. Sessions provided by a trained multidisciplinary team included informative talks and written material with dietary patterns, shopping lists, meal plans, and recipes. Individuals received personalized advice to increase the consumption of vegetables, fruits, legumes, and fish, as well as recommendations to reduce the intake of fast food, butter, sugar-sweetened beverages, and red or processed meats. In the CORDIOPREV study [25], the intervention group introduced increased plant-based protein intake with a Mediterranean diet, while in the PREDIMED [23] and PREDIMED-Reus [21] trials, members of the intervention group assigned to the two Mediterranean diet groups received allotments of either extra-virgin olive oil (50 mL/d) or mixed nuts (30 g/d: 15 g of walnuts, 7.5 g of almonds, and 7.5 g of hazelnuts) and either virgin olive oil (1 L/week) or mixed nuts (30 g/day), respectively. Participants in control groups following the low-fat diet were advised to decrease the consumption of all types of fat, including those from animal and plant sources. The risk of T2D showed significant association with the interventions compared to the control groups in all three studies; the HR was 0.6385 (95% CI: 0.4257–0.9587) in the CORDIOPREV [25], 0.70 (95% CI: 0.54–0.92) in the PREDIMED [23], and 0.48 (95% CI: 0.27–0.86) in the PREDIMED-Reus [21] study.

A total of 12 studies included a combination of diet and physical activity components enhanced by lifestyle counseling. The Finnish studies were based on the Finnish Diabetes Prevention Study (DPS), where overweight individuals (BMI ≥ 25) with impaired glucose tolerance (IGT) in the intervention group received personalized counseling to increase aerobic capacity and improve cardiorespiratory fitness. Individuals had the opportunity to participate in free-of-charge supervised exercise sessions in the gym [20]. Regarding changing dietary habits, to achieve individual goals, at least a 5% reduction in body weight, less than 30% of daily energy intake from fat, less than 10% of daily energy intake from saturated fat, and at least 15 g of fiber intake per 1000 kcal were set. Moreover, frequent ingestion of whole meal products, vegetables, berries and fruit, low-fat milk and meat products, soft margarines, and vegetable oils rich in monounsaturated fatty acids was recommended [18,19]. The interventions resulted in a significant reduction in T2D risk when compared to the control groups. The Finnish Diabetes Prevention Study resulted in a 58% overall reduction in the risk of T2D among participants aged 40–65 years with IGT (HR: 0.4, 95% CI: 0.3–0.7). The reduction in the incidence of T2D was associated with number and magnitude of lifestyle changes made [18,19]. The results from the extended follow-up of the Finnish DPS show that the effect of lifestyle intervention on diabetes risk did not disappear after active lifestyle counseling was stopped (HR: 0.57, 95% CI: 0.43–0.76) [19]. Lifestyle intervention in high-risk individuals had a long-lasting effect on the prevention of T2D with a relatively short active lifestyle intervention after a median follow-up time of 9 years (HR: 0.614, 95% CI: 0.478–0.789) [20].

Interventional studies from Spain [22,24] included intensive monthly educational sessions in small groups to promote healthy lifestyle changes, including healthy diet and physical activity among individuals with high risk of T2D. Participants in PreDE cluster RCT received continuous motivation through regular nurse-initiated phone calls every 6 weeks [24]. Educational programs for individuals with prediabetes or high risk of T2D were delivered over 6 h in two to four sessions to groups of five to fifteen participants. Topics covered general information on T2D and its risks, nutritional advice based on the PREDIMED MEDAS questionnaire to increase adherence to the Mediterranean diet, the benefits of physical activity, and tobacco cessation advice [22]. After a follow-up period of 2 years, a relative risk reduction of 32% (RR: 0.68, 95% CI: 0.47–0.99) was reported in the intervention group compared to the control in the PreDE cluster RCT [24], while the overall incidence of DM was reduced by 36% (HR: 0.54, 95% CI: 0.37–0.79) at the 4-year follow-up in the intervention group when the DE-PLAN-CAT public health program was applied [22].

Studies from the UK included the Norfolk Diabetes Prevention Study [30], where the intervention was delivered by trained health care professionals, aimed to support physical activity and dietary changes using patient-centered counseling. Behavior change targets were set by participants, including a 7% weight loss for those with BMI > 30, 150 min of moderate-intensity physical activity per week, muscle-strengthening exercises, and reduced fat intake. The counseling program comprised six two-hour group sessions over 12 weeks, targeted towards individuals at increased risk for T2D. Following the conclusion of the initial 12-week period, participants had up to 15 additional sessions scheduled every 8 weeks, beginning in the fourth month. After a median follow-up period of 2 years, a significant reduction in the risk of progressing to T2D among participants with high risk was observed in the intervention groups (OR: 0.57, 95% CI: 0.38–0.87) [30]. In the European Diabetes Prevention RCT in Newcastle upon Tyne [26], participants with impaired glucose tolerance in the intervention group met with a dietician and physiotherapist for 30 min sessions immediately post-randomization, then bi-weekly, monthly for three months, and every three months up to five years. They received an information pack on physical activity opportunities, a City Card, and an induction session with a trainer at a local leisure center. After a median follow-up of 3.1 years, a nonsignificant 55% reduction in DM incidence was observed in the intervention group (RR: 0.45, 95% CI: 0.2–1.2) [26]. In a 2-year RCT in the UK [29], all participants identified with prediabetes through the NHS Health Check program received personalized education on healthy diet and physical activity benefits at baseline. The intervention group additionally received 2–3 weekly SMS messages about lifestyle. Physical activity and SMS acceptability were monitored at baseline and follow-up. At the 2-year follow-up, there was no significant difference in the incidence of T2D between the intervention and control group (HR: 0.99, 95% CI: 0.69–1.43) [29]. The Let’s Prevent Diabetes cluster RCT [28] was a 6 h program delivered to groups of ten in either one full-day or two half-day sessions by two trained educators. Goals included a 5% body weight reduction, limiting fat intake, increasing fiber intake, and promoting physical activity. Participants had 3 h refresher sessions at 12 and 24 months to reinforce key messages and update action plans. After a follow-up period of 3 years, there was a nonsignificant 26% reduced risk of developing T2D (HR 0.74, 95% CI: 0.48–1.1) in the intervention group among individuals with prediabetes [28]. In a family-cluster randomized controlled trial [27], families received 15 dietitian visits over 3 years to achieve weight loss through a calorie-deficit diet and daily brisk walking. Culturally adapted resources and the Counterweight Program were used. Annual group sessions, including a food shopping tour and walking, were provided. Pedometers were given for motivation and progress assessment. With a 3-year follow-up, progression to T2D was less frequently observed in the intervention group compared to the control, but the difference was not significant (OR: 0.68, 95% CI: 0.27–1.67) [27].

Two studies from the Netherlands [31,32] investigated the effect of a lifestyle intervention on the incidence of T2D. Intervention in the SLIM study [31] included components focusing on diet and physical activity. The dietary recommendations followed the Dutch Nutrition Council’s guidelines for a healthy diet. Based on a 3-day physical activity log, participants received personalized advice from the researcher and/or a dietitian on how to increase their physical activity to at least 30 min a day, five days a week. In the randomized controlled trial carried out in Dutch primary care [32], 11 consultations, each lasting 20 min, were scheduled alternately with the nurse practitioner and the general practitioner. Additionally, five group meetings were organized by dietitians and physiotherapists to offer more detailed information on diet and exercise. Participants in the intervention group were also invited to a 1 h consultation with a dietitian, during which a 3-day food record was reviewed. After a mean follow-up of 4.1 years, a significant risk reduction in T2D was observed in the SLIM study among participants in the intervention group (RR: 0.53, 95% CI: 0.29–0.97) [31], while the cumulative incidence of T2D after 2.5 years was not significantly different between the intervention and control group in the Dutch primary care trial [32].

In a large pre-randomized general population study conducted in Denmark [17], researchers examined the effect of repeated general health checks on the 30-year incidence of T2D. The participants, aged 30, 40, 50, and 60, were from 11 municipalities in Copenhagen. The intervention group was invited to participate in up to three surveys between 1982 and 1994. Participants received personalized information about their results, disease risk, and lifestyle based on their responses and test results. Additionally, their general practitioners were provided with written summaries of the test results. Overall, the intervention group underwent repeated health checks and received tailored feedback and counseling based on their health assessments. The results indicate that the intervention had no beneficial effect on the development of DM (HR: 1.07, 95% CI: 0.98–1.16) [17] (Table 4).

## 4. Discussion

This systematic review represents the first analysis of the interventions implemented and evaluated in the EU-28 with the aim of preventing or delaying T2D in healthy and high-risk individuals. Compared with the control conditions, 10 studies reported a significant reduction in the incidence of T2D after completing the intervention [18,19,20,21,22,23,24,25,30,31], while 6 studies showed a nonsignificant association between the interventions and outcome [26,27,28,29,32,33].

When interventions included dietary and physical activity components supplemented by individual guidance on T2D risk management, individually tailored goal settings were effective in reducing the T2D risk. Nevertheless, modifications to dietary habits were also found to be effective. Long-term adherence to a Mediterranean diet, supplemented with extra-virgin olive oil, mixed nuts, or plant-based proteins, without energy restrictions, resulted in a substantial reduction in the risk of T2D among older persons with high cardiovascular risk [21,22,23]. Lifestyle interventions aimed at weight reduction, a healthy diet, and increased physical activity in high-risk individuals had a long-lasting effect in the prevention of the disease among both women and men [20]. Early, comprehensive lifestyle change as the primary target of a T2D prevention strategy with a relatively short active lifestyle intervention was able to extend time free of DM [31], but the effects were limited to those who had high-risk glycemic categories [18]. Our findings support existing evidence from EU investigations that multicomponent interventions, which include dietary behaviors, physical activity elements, and personalized lifestyle counseling, are effective in reducing the risk of T2D among healthy and high-risk individuals. Large effects were found when a high-risk approach was applied at the individual level, especially among people with prediabetic conditions. However, when the interventions were delivered at the population level, the effects were smaller and less significant [17].

Our systematic review highlights key differences between interventions that significantly reduced the incidence of T2D and those that did not. Interventions combining dietary modifications, physical activity, and personalized lifestyle counseling were generally more effective. For example, the Finnish Diabetes Prevention Study and the PREDIMED trials demonstrated significant reductions in T2D risk through comprehensive lifestyle changes and adherence to the Mediterranean diet. In contrast, interventions lacking these multifaceted approaches often failed to show significant benefits. For instance, a Danish study focusing solely on repeated health checks with personalized feedback did not yield significant reductions in T2D incidence. Similarly, interventions relying on minimal interventions, such as SMS messages for lifestyle advice, also did not demonstrate significant effects. These findings suggest that the effectiveness of interventions may depend on their comprehensiveness and the level of personalized support provided. Multicomponent interventions tailored to individual needs and incorporating both dietary and physical activity elements were more likely to achieve significant reductions in T2D incidence.

A population-wide prevention strategy is often more straightforward to implement, as it obviates the need to identify and target high-risk individuals. Such a strategy may also be more efficacious because it does not necessitate behavior modification and can be sustained indefinitely. This raises pertinent questions regarding the equity and efficacy of different prevention approaches. A simulation study assessing the impact of primary and secondary interventions has elucidated the differential effects of population versus high-risk focused interventions. The findings indicate that population-wide secondary prevention interventions yield the most significant overall reductions in both the standard deviation and prevalence of the disease. Conversely, high-risk focused primary prevention interventions targeting major risk factors result in substantial reductions in both the prevalence and standard deviation of the disease within the high-risk cohort.

Evidence from the reviewed studies indicates that interventions combining dietary improvements and physical activity, supported by personalized lifestyle counseling, are particularly effective in reducing the risk of developing T2D. Specifically, adherence to a Mediterranean diet rich in extra-virgin olive oil, nuts, and plant-based proteins without caloric restriction has been shown to significantly lower T2D risk among older individuals with high cardiovascular risk. Additionally, comprehensive lifestyle interventions aimed at weight loss and the adoption of healthy dietary and exercise habits have demonstrated long-term benefits in both men and women at high risk of T2D. These findings underscore the importance of the multifaceted approach to lifestyle modification, emphasizing personalized, culturally sensitive guidance to enhance adherence and effectiveness across diverse populations [20,36,37,38,39,40].

Community-based educational interventions play a crucial role in preventing T2D. These programs improve knowledge, self-efficacy, and self-care practices among individuals, leading to positive health outcomes. Previous research has shown their effectiveness by demonstrating the intervention’s ability to improve self-care concerning glycemic control [41]. Further evidence comes from a systematic review and meta-analysis, which shows these interventions can significantly reduce T2D incidence by 46% [42]. Successful implementation of these interventions necessitates a fully engaged, collaborative approach to planning and execution [43]. The results found in the present study align with previous studies that found lifestyle interventions, such as diet modification and physical activity promotion programs, could significantly reduce the risk of T2D in adults even if implemented at a low to moderate frequency. For large populations with scarce resources, a lower frequency of lifestyle interventions could be effective, but the program duration must be at least 12 months [44]. It is crucial to enhance the knowledge of healthcare providers about prediabetes treatment and management. This should involve training in identifying recommended lifestyle modifications, clinical targets, and pharmacological therapies for patients with prediabetes [45]. The findings of this study highlight the necessity of increasing access to lifestyle intervention programs to avoid the development of T2D. Previous research has found barriers like cost, availability, and awareness that can limit participation [46]. An integrated healthcare system that works with politicians is vital for lowering program fees, increasing the number of intervention sites, and conducting focused outreach initiatives to raise awareness. This is consistent with the success factors identified by a prior study that used the PIPE (penetration, implementation, participation, and effectiveness) impact metric [47], a program penetration component that works through successful strategies to reach target populations, such as mail invitations, media advertising, and contacting local physicians or churches [44]. To ensure that these interventions are implemented successfully, healthcare providers must be knowledgeable about prediabetes and its management. It is critical to emphasize that a prediabetes diagnosis represents an opportunity to prevent or delay the onset of T2D [48].

Providers should be educated on the importance of clear communication about the high risk of disease progression, even though effective treatments exist to prevent or delay such progression. There are some strategies for educating healthcare providers about prediabetes treatment. First, the National Diabetes Prevention Program (National DPP) [49] encourages providers to refer prediabetic patients to the National DPP. This lifestyle change program is led by qualified coaches and it significantly lowers the risk of T2D. Second, Diabetes Self-Management Education and Support (DSMES) [50], in which providers play an important role in increasing access to DSMES, teaches patients practical skills for managing DM in everyday life. Finally, providers must stay up to date with the latest Standards of Diabetes Care; these guidelines cover screenings, management, and related comorbidities and regularly review and implement evidence-based practices in the clinical setting [51].

Our study has some limitations that must be acknowledged. First, studies from only five countries were identified—Finland, Denmark, Spain, the Netherlands, and the UK—which limits the generalizability of our findings to the entire EU. Second, the studies spanned only an 18-year period from 2003 to 2021, excluding more recent data and any data prior to 2003, potentially affecting the comprehensive understanding of long-term intervention impacts. Additionally, only one of the included studies was of “high quality”, while the remaining fifteen were of “moderate quality” or “low quality”, which may compromise the reliability of our conclusions. The heterogeneity in interventions, methods, and reported outcomes precluded us from performing a meta-analysis, thereby limiting our ability to synthesize results quantitatively. Furthermore, the varied reporting formats of T2D incidence data—such as incidence rates, hazard ratios, risk ratios, and odds ratios—affected direct comparisons and synthesis of findings. Although each intervention included a control group, the method of randomization and the statistical power often limited the reliability of the outcome results. Despite these limitations, our study possesses several strengths. It is a novel systematic review that evaluates the effect of interventions on T2D incidence prospectively including data from EU member states. Importantly, all included studies were interventional, with the majority being RCTs, and each had a control group, enhancing the robustness of our findings. Additionally, our study collected subregional data, enabling comparisons not only at the national level but also across specific regions. Another significant strength lies in the rigorous methodology employed throughout the selection and evaluation process, ensuring a thorough and systematic approach to data analysis. The methodological rigor increases the credibility and relevance of our study’s outcomes.

## 5. Conclusions

This review found that there are only a few scientific publications that report primary lifestyle interventions with the outcome of T2D incidence. The review addressed two key research questions related to the primary prevention of T2D among nondiabetic populations in the EU-28. First, regarding the effectiveness of preventive interventions compared to standard care, the findings indicate that lifestyle modifications, particularly those combining dietary changes and physical activity with personalized counseling, significantly reduce T2D incidence in high-risk individuals. However, the impact among low-risk populations remains limited and less generalizable. Second, when comparing different preventive measures, interventions such as adherence to a Mediterranean diet and structured weight loss programs demonstrated notable effectiveness in lowering T2D risk, particularly among individuals with prediabetes or other high-risk glycemic categories. Multicomponent strategies integrating diet, physical activity, and tailored counseling proved most effective, underscoring the importance of personalized approaches in prevention efforts. Further large-scale randomized studies targeting both high- and low-risk populations are essential to establish reliable evidence for population-wide strategies that can effectively reduce T2D incidence across diverse groups.

To obtain reliable and generalizable results, further large-scale, properly randomized interventional studies are needed, where the primary outcome is the change in the incidence of T2D, with an adequate follow-up period. Furthermore, interventional studies are needed among low-risk individuals to see how studies using various population-based intervention approaches affect the development of T2D over the years. The aforementioned knowledge is of the utmost importance to the formulation of sound policy.

## Figures and Tables

**Table 1:** Main characteristics of the included studies.

Title	Author (Year)	Country	City or Region	Study Design	Sample (n)	Male (n)	Female (n)	Total Sample Age Range	Follow-Up Time
A randomized general population study of the effects of repeated health checks on incident diabetes	Skaaby et al. (2018) [17]	Denmark	Suburbs of Copenhagen	Randomized trial (pre-randomized allocation of participants)	17,631 Control: 12,892 Intervention: 4739	8700 Control: 6333 Intervention: 2367	8931 Control: 6559 Intervention: 2372	30 to 60 years old	24.1 years (mean)
Prevention of diabetes mellitus in subjects with impaired glucose tolerance in the Finnish Diabetes Prevention Study: results from a randomized clinical trial	Lindström et al. (2003) [18]	Finland	Helsinki, Kuopio, Oulu, Tampere, and Turku	Randomized controlled trial	522 Control: 257 Intervention: 265	172 Control: 81 Intervention: 91	350 Control: 176 Intervention: 174	40 to 65 years old 55 ± 7	3.2 years (mean)
Sustained reduction in the incidence of type 2 diabetes by lifestyle intervention: follow-up of the Finnish Diabetes Prevention Study	Lindström et al. (2006) [19]	Finland	Helsinki, Kuopio, Oulu, Tampere, and Turku	Randomized controlled trial	522 Control: 257 Intervention: 265	172 Control: 31% Intervention: 34%	350 Control: 69% Intervention: 66%	40 to 64 years old 55 (mean)	7 years (median)
Improved lifestyle and decreased diabetes risk over 13 years: long-term follow-up of the randomized Finnish Diabetes Prevention Study (DPS)	Lindström et al. (2012) [20]	Finland	Helsinki, Kuopio, Oulu, Tampere, and Turku	Randomized controlled trial	522 Control: 257 Intervention: 265	172	350	40 to 64 years old 55 ± 7	0–16 years 9 years (median)
Reduction in the incidence of type 2 diabetes with the Mediterranean diet: results of the PREDIMED-Reus nutrition intervention randomized trial	Salas-Salvadó et al. (2011) [21]	Spain	Northeastern Spain	Multicenter, randomized, parallel-group primary prevention trial	418 Control: 134 Intervention group 1: 139 Intervention group 2: 145	175 Control: 51 Intervention 1: 56 Intervention 2: 68	243 Control: 83 Intervention group 1: 83 Intervention group 2: 77	55 to 80 years old Control: 67.8 ± 6.1 Intervention group 1: 67.4 ± 6.1 Intervention group 2: 66.6 ± 5.8	4 years (median)
Delaying progression to type 2 diabetes among high-risk Spanish individuals is feasible in real-life primary healthcare settings using intensive lifestyle intervention	Costa et al. (2012) [22]	Spain	Catalonia	Prospective cohort study	552 Control: 219 Intervention: 333	184 Control: 78 Intervention: 106	368 Control: 141 Intervention: 227	45 to 75 years old Control: 62.0 ± 7.9 Intervention group: 62.2 ± 8.0	3.8 years (mean) 4.2 years (median)
Prevention of diabetes with Mediterranean diets: a subgroup analysis of a randomized trial	Salas-Salvadó et al. (2014) [23]	Spain	N/A	Parallel-group, randomized, primary cardiovascular prevention trial	3541 Control: 1147 Intervention group 1: 1154 Intervention group 2: 1240	1346 Control: 401 Intervention group 1: 439 Intervention group 2: 5,061,346	2195 Control: 746 Intervention group 1: 715 Intervention group 2: 734	55 to 80 years old Control: 66.5 ± 6.0 Intervention group 1: 66.2 ± 6.0 Intervention group 2: 67.2 ± 6.1	4.1 years (median)
Effective translation of a type-2 diabetes primary prevention program into routine primary care: the PreDE cluster randomized clinical trial	Sanchez et al. (2018) [24]	Spain	The Basque country	Phase IV randomized cluster clinical trial	1088 Control: 634 Intervention: 454	418 Control: 266 Intervention: 152	670 Control: 368 Intervention: 302	45 to 70 years old 59.3 ± 6.9	2 years
Quality and quantity of protein intake influence incidence of type 2 diabetes mellitus in coronary heart disease patients: from the CORDIOPREV study	de la Cruz-Ares et al. (2021) [25]	Spain	Córdoba	Randomized, single-blind, controlled intervention trial	436 Control: 218 Intervention: 218	369 Control: 182 Intervention: 187	67 Control: 36 Intervention: 31	20 to 76 years old Control: 57.4 ± 0.6 Intervention: 58.1 ± 0.6	5 years (median)
Prevention of type 2 diabetes in adults with impaired glucose tolerance: the European Diabetes Prevention RCT in Newcastle upon Tyne, UK	Penn et al. (2009) [26]	UK	Newcastle upon Tyne	Randomized controlled trial	102 Control: 51 Intervention: 51	41 Control: 20 Intervention: 21	61 Control: 31 Intervention: 30	38 to 74 years old Control: 57.4 Intervention group: 56.8	0–5 years 3.1 years (mean)
Effect of a lifestyle intervention on weight change in south Asian individuals in the UK at high risk of type 2 diabetes: a family-cluster randomized controlled trial	Bhopal et al. (2014) [27]	UK	Edinburgh or Glasgow (Scotland)	Non-blinded, family-cluster randomized controlled trial	171 Control: 86 Intervention: 85	78 Control: 39 Intervention: 39	93 Control: 46 Intervention: 47	35 to 80 years old Control: 52.2 ± 10.3 Intervention group: 52.8 ± 10.2	3 years
A community-based primary prevention program for type 2 diabetes integrating identification and lifestyle intervention for prevention: the Let’s Prevent Diabetes cluster randomized controlled trial	Davies et al. (2016) [28]	UK	Leicestershire	Cluster randomized controlled trial	880 Control: 433 Intervention: 447	560 Control: 278 Intervention: 282	320 Control: 155 Intervention: 165	40–75 if White European; 25–75 years if South Asian Control: 63.9 ± 7.9 Intervention group: 63.9 ± 7.6	3 years (median)
A pragmatic and scalable strategy using mobile technology to promote sustained lifestyle changes to prevent type 2 diabetes in India and the UK: a randomized controlled trial	Nanditha et al. (2020) [29]	UK	N/A	Randomized controlled trial	891 Control: 444 Intervention: 447	432 Control: 216 Intervention: 216	459 Control: 228 Intervention: 231	40 to 74 years old Control: 60.2 ± 9.3 Intervention group: 60.3 ± 9.4	2 years
Lifestyle intervention with or without lay volunteers to prevent type 2 diabetes in people with impaired fasting glucose and/or nondiabetic hyperglycemia: a randomized clinical trial	Sampson et al. (2021) [30]	UK	East of England	Randomized clinical 3-arm parallel-group trial	1028 Control: 178 Intervention group 1: 424 Intervention group 2: 426	645 Control: 108 Intervention group 1: 258 Intervention group 2: 279	383 Control: 70 Intervention group 1: 166 Intervention group 2: 147	Above 40 years old Control: 65.3 ± 10 Intervention group 1: 66.5 ± 8.6 Intervention group 2: 66.7 ± 9.5	3.8 years 2 years (mean)
Predictors of lifestyle intervention outcome and dropout: the SLIM study	Roumen et al. (2011) [31]	The Netherlands	Maastricht	Randomized controlled trial	147 Control: 73 Intervention: 74	75 Control: 37 Intervention: 38	72 Control: 36 Intervention: 36	Control: 58.8 ± 8.4 Intervention group: 55.0 ± 6.5	3–6 years 4.1 years (mean)
A lifestyle intervention to reduce Type 2 diabetes risk in Dutch primary care: 2.5-year results of a randomized controlled trial	Vermunt et al. (2012) [32]	The Netherlands	Eindhoven and surrounding	Randomized controlled trial	925 Control: 446 Intervention: 479	N/A	N/A	40 to 70 years old	2.5 years

**Table 2:** Quality assessment of the included interventional studies was conducted utilizing the NHLBI quality assessment tool for controlled intervention studies [16].

References	Q1	Q2 *	Q3	Q4	Q5	Q6 *	Q7	Q8	Q9 *	Q10	Q11 *	Q12	Q13	Q14	Quality
Skaaby et al. [17]	Y	NR	N	NA	NR	Y	CD	CD	NR	NR	NR	NR	Y	Y	L
Lindström et al. [20]	Y	NR	N	NA	N	Y	N	Y	Y	NA	Y	N	Y	Y	L
de la Cruz-Ares et al. [25]	Y	Y	NR	NA	N	Y	NR	NR	NR	NR	Y	NR	N	Y	L
Vermunt et al. [32]	Y	N	NR	NA	N	Y	N	Y	NR	NR	Y	Y	Y	NR	L
Sanchez et al. [24]	Y	Y	N	NA	Y	Y	Y	Y	NR	Y	Y	Y	Y	Y	M
Sampson et al. [30]	Y	Y	Y	NA	NR	Y	N	N	NR	Y	Y	Y	Y	Y	L
Salas-Salvadó et al. [23]	Y	Y	Y	NA	Y	Y	Y	Y	Y	Y	Y	Y	Y	Y	H
Salas-Salvadó et al. [21]	Y	NR	NR	NA	Y	Y	CD	CD	Y	Y	Y	NR	Y	Y	L
Roumen et al. [31]	Y	NR	NR	NA	NR	Y	N	Y	N	N	CD	CD	Y	Y	L
Penn et al. [26]	Y	Y	Y	NA	N	N	Y	N	CD	CD	Y	N	Y	Y	L
Nanditha et al. [29]	Y	Y	Y	NA	Y	Y	CD	CD	Y	NR	Y	CD	Y	Y	L
Lindström et al. [19]	Y	NR	NR	NA	NR	Y	Y	Y	N	NR	Y	CD	Y	Y	L
Lindström et al. [18]	Y	NR	NR	NA	NR	Y	Y	NR	CD	NR	CD	NR	Y	Y	L
Davies et al. [28]	Y	Y	Y	NA	CD	N	N	Y	CD	CD	Y	CD	Y	Y	L
Costa et al. [22]	N	NA	N	NA	CD	Y	N	Y	CD	N	Y	Y	CD	Y	L
Bhopal et al. [27]	Y	Y	Y	NA	N	Y	Y	Y	N	CD	Y	Y	Y	Y	M

Y: Yes; N: No; CD: Cannot determine; NA: Not applicable; NR: Not reported. H: high quality; M: moderate quality; L: low quality. Assessment questions: Q1: Was the study described as randomized, a randomized trial, a randomized clinical trial, or an RCT? Q2: Was the method of randomization adequate (i.e., use of randomly generated assignment)? Q3: Was the treatment allocation concealed (so that assignments could not be predicted)? Q4: Were study participants and providers blinded to treatment group assignment? Q5: Were the people assessing the outcomes blinded to the participants’ group assignments? Q6: Were the groups similar at baseline on important characteristics that could affect outcomes (e.g., demographics, risk factors, comorbid conditions)? Q7: Was the overall drop-out rate from the study at endpoint 20% or lower of the number allocated to treatment? Q8: Was the differential drop-out rate (between treatment groups) at endpoint 15 percentage points or lower? Q9: Was there high adherence to the intervention protocols for each treatment group? Q10: Were other interventions avoided or similar in the groups (e.g., similar background treatments)? Q11: Were outcomes assessed using valid and reliable measures implemented consistently across all study participants? Q12: Did the authors report that the sample size was sufficiently large to be able to detect a difference in the main outcome between groups with at least 80% power? Q13: Were the outcomes reported or the subgroups analyzed prespecified (i.e., identified before analyses were conducted)? Q14: Were all randomized participants analyzed in the group to which they were originally assigned, i.e., did they use an intention-to-treat analysis? *: Highly critical domain items 2, 6, 9, and 11.

**Table 3:** Additional characteristics of the included studies.

Author (Year)	Ethical Approval	Ethical Approval Details	Type 2 Diabetes Diagnosis	Comorbidity	Population Characteristics
Skaaby et al. (2018) [17]	Yes	The study was conducted according to the Helsinki Declaration and Danish relevant ethics committees. All participants gave their written and informed consent.	Hospitalization with a diagnosis of diabetes; measurements of blood glucose either five times or more within 1 year; two or more annual measurements during a 5-year period; registered use of chiropody (coded for diabetes); prescription of oral antidiabetic medication or insulin at least twice	Not reported	Danish people age 30–60 without type 2 diabetes from 11 municipalities in the suburbs of Copenhagen
Lindström et al. (2003) [18]	Yes	The ethics committee of the National Public Health Institute in Helsinki, Finland, approved the study protocol. All study participants gave written informed consent.	Defined according to the WHO 1985 criteria [34], i.e., either a fasting plasma glucose concentration over 140 mg/dL or 2 h post-challenge plasma glucose concentration of 200 mg/dL	Overweight subjects, body mass index (BMI) > 25 kg/m^2^ and with IGT	Finnish overweight subjects, body mass index (BMI) > 25 kg/m^2^ with IGT aged 40 to 65 years
Lindström et al. (2006) [19]	Yes	The ethics committee of the National Public Health Institute in Helsinki, Finland, approved the study protocol. All study participants gave written informed consent.	Diabetes was defined according to WHO 1985 criteria, i.e., either fasting plasma glucose of 7.8 mmol/L or more or 2 h post-challenge plasma glucose of 11.1 mmol/L or more	Overweight, middle-aged men and women with impaired glucose tolerance	Overweight (mean body mass index 31.1 kg/m^2^), middle-aged (mean age 55 years) participants with impaired glucose tolerance
Lindström et al. (2012) [20]	Yes	The ethics committees of the National Public Health Institute in Helsinki, Finland (intervention phase), and of the North Ostrobothnia Hospital District (follow-up period) approved the study protocol. All study participants gave written informed consent at baseline and again at the beginning of the post- intervention follow-up	Fasting plasma glucose ≥ 7.8 mmol/L or 2 h plasma glucose ≥ 11.1 mmol/L in two separate OGTTs (WHO 1985 criteria)	Overweight, middle-aged persons with impaired glucose tolerance	European population aged between 40 and 64, overweight persons with impaired glucose tolerance
Salas-Salvadó et al. (2011) [21]	Yes	The local institutional review board approved the study protocol. All participants provided written informed consent.	American Diabetes Association criteria: fasting plasma glucose > 7.0 mmol/L or 2 h plasma glucose > 11.1 mmol/L after a 75 g oral glucose load, measured yearly	At least three cardiovascular risk factors, namely smoking, hypertension, dyslipidemia, overweightness (BMI ≥ 25 kg/m^2^), and family history of premature cardiovascular disease (55 years in men and 60 years in women)	Community-dwelling men and women without prior cardiovascular disease but with at least three cardiovascular risk factors and family history of premature cardiovascular disease
Costa et al. (2012) [22]	Yes	The research ethics committee board at the Jordi Gol Research Institute (Barcelona, Spain) approved the study protocol. All participants gave written informed consent.	OGTT > 11.1 mmol/L (WHO criteria)	FINDRISC score >14 or prediabetes defined using WHO criteria for fasting or 2 h glucose	White European individuals without diabetes aged 45–75 years
Salas-Salvadó et al. (2014) [23]	Yes	The institutional review board (IRB) of Hospital Clinic (Barcelona, Spain) approved the study protocol. All participants provided written informed consent.	American Diabetes Association (ADA) criteria: fasting plasma glucose levels of 7.0 mmol/L or greater (126.1 mg/dL) or 2 h plasma glucose levels of 11.1 mmol/L or greater (200.0 mg/dL) after a 75 g oral glucose load	Community-dwelling men and women without CVD at baseline who had either type 2 diabetes or at least three or more cardiovascular risk factors, namely current smoking, hypertension, hypercholesterolemia, low high-density lipoprotein cholesterol levels, overweightness or obesity, and family history of premature CVD	Spanish men and women without diabetes at high cardiovascular risk
Sanchez et al. (2018) [24]	Yes	The Basque Country Clinical Research Ethics Committee approved the study protocol. All participants received the patient information sheet and gave written consent.	Assessed by the OGTT in accordance with the WHO protocol and a cut-off point for type 2 diabetes diagnosis ≥ 200 mg/mL	Attendees considered at high risk of T2D (FINDRISC ≥ 14 points)	Spanish nondiabetic 45 to 70-year-old primary health care attendees considered at high risk of type 2 diabetes (FINDRISC ≥ 14 points)
de la Cruz-Ares et al. (2021) [25]	Yes	The local ethics committees, the Helsinki Declaration, and Good Clinical Practice guidelines approved the trial protocol. All participants gave written informed consent to participate in the study.	Fasting glucose ≥ 126 mg/dL, 2 h glucose during OGTT ≥ 200 mg/dL, or HbA1c ≥ 6.5% (ADA criteria for diagnosis of type 2 diabetes)	Coronary heart disease patients of European ancestry who had their last coronary event more than 6 months before enrollment	Coronary heart disease patients of European ancestry who had their last coronary event more than 6 months before enrollment
Penn et al. (2009) [26]	Yes	The Newcastle and North Tyneside NHS Research Ethics Committee approved the study protocol. All participants gave informed, written consent before the start of the study.	Derived from the mean of two standard oral glucose tolerance tests (OGTTs)—stratum 1: 7.8 to 9.4 mmol/L; stratum 2: 9.5 to 11.1 mmol/L)	Impaired glucose tolerance	Participants from the UK, aged between 38 and 74
Bhopal et al. (2014) [27]	Yes	The Scotland A Research Ethics Committee approved the study. Outcomes were reviewed annually by a data monitoring and ethics committee.	75 g oral glucose tolerance test. The oral glucose tolerance test followed standardized procedures, with venous blood taken after an overnight fast of 10–16 h and 2 h after glucose load	Impaired glucose tolerance or impaired fasting glucose	Individuals of south Asian descent living in the UK
Davies et al. (2016) [28]	Yes	Ethical approval was sought, and the study involved practice-level and individual-level informed consent.	WHO 1999 [34] criteria/guidelines. Following the update of the WHO diagnostic criteria to include HbA1c (WHO, 2011) [35], we obtained a protocol amendment in January 2013, allowing HbA1c ≥ 6.5% to become part of the diagnostic criteria for T2DM within this study	people at risk of PDM/T2DM	people with prediabetes mellitus
Nanditha et al. (2020) [29]	Yes	In the UK, approval was from the Westminster Research Ethics Committee and site specific assessment (SSA) plus research and development (R&D) approvals were in place at each participating NHS Trust.	Diabetes as defined by international criteria for fasting plasma glucose or HbA1c at any study review visit or in any healthcare setting	People with prediabetes defined by an HbA1c level of ≥42 and ≤47 mmol/mol (≥6.0% and ≤6.4%	People who met the HbA1c entry criteria (≥42 and ≤47 mmol/mol [≥6.0% and ≤6.4%])
Sampson et al. (2021) [30]	Yes	The National Research Ethics Service research ethics committee approved the study. All participants gave written informed consent.	Paired fasting glucose measurements both ≥ 7.0 mmol/L, or a 2 h OGTT ≥ 11.1 mmol/L undertaken if HbA1c ≥ 42 mmol/L in IFG participants (WHO criteria)	People at increased risk of type 2 diabetes: elevated fasting plasma glucose level alone or an elevated glycated hemoglobin level with an elevated fasting plasma glucose level/people with current prediabetes glycemic categories	People with nondiabetic hyperglycemia
Roumen et al. (2011) [31]	Yes	The Medical Ethics Review Committee of Maastricht University approved the study protocol. All subjects gave written informed consent before the start of the study.	The incidence of type 2 diabetes was determined according to the World Health Organization criteria of 1999	Impaired glucose tolerance	Dutch population with impaired glucose tolerance (IGT)
Vermunt et al. (2012) [32]	Yes	The Medical Ethical Review Committee of the Catharina Hospital in Eindhoven, the Netherlands, approved the study.	Diagnosis of type 2 diabetes was based on one oral glucose tolerance test according to the 2006 World Health Organization diagnostic criteria	Participants with FINDRISC score ≥ 13 (higher risk of developing type 2 diabetes)	European, aged between 40 and 70

**Table 4:** Outcomes from the included studies.

Title	Author (Year)	Country	Intervention	Number of Cases (n)	Value of the Outcome Measure (Control)	Value of the Outcome Measure (Intervention)	Interpretation
A randomized general population study of the effects of repeated health checks on incident diabetes	Skaaby et al. (2018) [17]	Denmark	Repeated general health checks. Personalized feedback and counseling based on their health assessments.	2636	HR: 1.07 (95% CI: 0.98–1.16, *p* = 0.153)		Repeated general health checks with personalized feedback and counseling showed no beneficial and significant intervention effect on the development of diabetes.
Prevention of diabetes mellitus in subjects with impaired glucose tolerance in the Finnish Diabetes Prevention Study: results from a randomized clinical trial	Lindström et al. (2003) [18]	Finland	Modifying eating behavior. Seven sessions with a nutritionist during the first year of the study and every 3 mo thereafter. Individually tailored circuit-type resistance training sessions.	Control: 59 Intervention: 27	Cumulative incidence percentage (Year 1): 6.1 (95% CI: 3.2–9.0) Cumulative incidence percentage (Year 2): 14.4 (95% CI: 9.9 to 18.6) Cumulative incidence percentage (Year 3): 20.9 (95% CI: 15.5 to 25.9) Cumulative incidence percentage (Year 4): 23.0 (95% CI: 16.9 to 28.6) Cumulative incidence percentage (Year 5): 34.4 (95% CI: 21.9 to 44.9) Cumulative incidence percentage (Year 6): 42.6 (95% CI: 26.0 to 55.5)	Cumulative incidence percentage (Year 1): 1.9 (95% CI: 0.2 to 3.6) Cumulative incidence percentage (Year 2): 6.3 (95% CI: 3.2 to 9.2) Cumulative incidence percentage (Year 3): 9.1 (95% CI: 5.4 to 12.6) Cumulative incidence percentage (Year 4): 10.9 (95% CI: 6.4 to 15.2) Cumulative incidence percentage (Year 5): 20.0 (95% CI: 8.8 to 29.8) Cumulative incidence percentage (Year 6): 20.0 (95% CI: 8.8 to 29.8)	The risk of diabetes was reduced by a statistically significant 58% in the intervention group compared to the control.
					HR: 0.4 (95% CI: 0.3–0.7, *p* < 0.001)		
Sustained reduction in the incidence of type 2 diabetes by lifestyle intervention: follow-up of the Finnish Diabetes Prevention Study	Lindström et al. (2006) [19]	Finland	Intensive diet-exercise counseling. Detailed and individualized counseling to achieve the lifestyle goals. Individually tailored circuit-type moderate-intensity resistance training sessions.	Control: 110 Intervention: 75	Incidence rate per 100 person-years: 7.4 (95% CI: 6.1–8.9, *p* = 0.001)	Incidence rate per 100 person-years: 4.3 (95% CI: 3.4–5.4, *p* = 0.001)	The overall incidence of diabetes was reduced by a statistically significant 43% in the intervention group compared to the control.
					HR: 0.57 (95% CI: 0.43–0.76, *p* = 0.001)		
Improved lifestyle and decreased diabetes risk over 13 years: long-term follow-up of the randomized Finnish Diabetes Prevention Study (DPS)	Lindström et al. (2012) [20]	Finland	Individualized lifestyle intervention. Counseling sessions with nutritionist. Supervised exercise sessions in the gym.	Control: 140 Intervention: 106	Incidence rate per 100 person-years (control): 7.2 (95% CI 6.1–8.5) Incidence rate per 100 person-years (intervention): 4.5 (95% CI 3.8–5.5) HR: 0.614 (95% CI: 0.478–0.789, *p* < 0.001)		Absolute risk of developing type 2 diabetes was reduced by a statistically significant 19.4% in the intervention group compared to the control.
Reduction in the incidence of type 2 diabetes with the Mediterranean diet: results of the PREDIMED-Reus nutrition intervention randomized trial	Salas-Salvadó et al. (2011) [21]	Spain	Behavioral intervention promoting the MedDiet. Allocated diet (low-fat or Med diet).	Control: 24 Intervention group 1: 14 Intervention group 2: 16	Incidence rate per 1000 person-years: 46.4 (95% CI: 30.1–68.5) Cumulative incidence rate: 17.9 (95% CI: 11.4–24.2)	Incidence rate per 1000 person-years (MedDiet + VOO): 24.6 (95% CI: 13.5–40.8) Incidence rate per 1000 person-years (MedDiet + Nuts): 26.8 (95% CI: 15.3–43.0) Cumulative incidence rate (MedDiet + VOO): 10.1 (95% CI: 5.1–15.1) Cumulative incidence rate (MedDiet + Nuts): 11.0 (95% CI: 5.9–16.1)	Following a median follow-up of 4.0 years, diabetes rates were reduced by a statistically significant 51% and 52% with the consumption of a Mediterranean diet supplemented with virgin olive oil or mixed nuts, respectively, compared to the control diet group.
					HR crude (MedDiet + VOO vs. Con): 0.53 (95% CI: 0.27–1.09) HR crude (MedDiet + Nuts vs. Con): 0.58 (95% CI: 0.31–1.10) HR crude (Combined MedDiet vs. Con): 0.55 (95% CI: 0.32–0.95) HR age- and sex-adjusted (MedDiet + VOO vs. Con): 0.52 (95% CI: 0.27–1.00) HR age- and sex-adjusted (MedDiet + Nuts vs. Con): 0.55 (95% CI: 0.29–1.00) HR age- and sex-adjusted (Combined MedDiet vs. Con): 0.53 (95% CI: 0.31–0.92) HR multivariate-adjusted (MedDiet + VOO vs. Con): 0.49 (95% CI: 0.25–0.97) HR multivariate-adjusted (MedDiet + Nuts vs. Con): 0.48 (95% CI: 0.24–0.96) HR multivariate-adjusted (Combined MedDiet vs. Con): 0.48 (95% CI: 0.27–0.86)		
Delaying progression to type 2 diabetes among high-risk Spanish individuals is feasible in real-life primary healthcare settings using intensive lifestyle intervention	Costa et al. (2012) [22]	Spain	A 6 h educational program scheduled in two to four sessions. Increase knowledge of type 2 diabetes risks. Promote the Mediterranean diet and provide nutritional advice. Increase adherence to the Mediterranean diet.	Control: 63 Intervention: 61	HR: 0.54 (95% CI: 0.37–0.79)		The risk of diabetes was reduced by a statistically significant 46% in the intervention group compared to the control.
					Incidence percentage: 22.8% (95% CI: 22.9–35.3%)	Incidence percentage: 18.3% (95% CI: 14.3–22.9%)	
					HR: 0.64 (95% CI: 0.47–0.87, *p* < 0.004)		
Prevention of diabetes with Mediterranean diets: a subgroup analysis of a randomized trial	Salas-Salvadó et al. (2014) [23]	Spain	Behavioral intervention promoting the Mediterranean diet. Allocated diet (low-fat or Med diet).	Control: 101 Intervention group 1: 80 Intervention group 2: 92	Incidence rate per 1000 person-years: 23.6 (95% CI: 19.3–28.7) Cumulative incidence: 8.81 (95% CI: 7.23–10.60)	Incidence rate per 1000 person-years (MedDiet + EVOO): 16 (95% CI: 12.7–19.9) Cumulative incidence (MedDiet + EVOO): 6.93 (95% CI: 5.53–8.55) Incidence rate per 1000 person-years (MedDiet + Nuts): 18.7 (95% CI: 15.1–22.9) Cumulative incidence (MedDiet + Nuts): 7.42 (95% CI: 6.02–9.02)	Following a median follow-up of 4.1 years, the Mediterranean diet groups supplemented with extra-virgin olive oil and mixed nuts showed a statistically significant 40% relative risk reduction and a nonsignificant 18% risk reduction in diabetes risk, respectively, compared to the control diet group.
					HR crude (MedDiet + EVOO vs. Con): 0.69 (95% CI: 0.51–0.92) HR crude (MedDiet + Nuts vs. Con): 0.81 (95% CI: 0.61–1.08) HR crude (Combined MedDiet vs. Con): 0.75 (95% CI: 0.58–0.96) HR age- and sex-adjusted (MedDiet + EVOO vs. Con): 0.68 (95% CI: 0.51–0.92) HR age- and sex-adjusted (MedDiet + Nuts vs. Con): 0.80 (95% CI: 0.60–1.06) HR age- and sex-adjusted (Combined MedDiet vs. Con): 0.74 (95% CI: 0.58–0.95) HR multivariate-adjusted A (MedDiet + EVOO vs. Con): 0.68 (95% CI: 0.51–0.92) HR multivariate-adjusted A (MedDiet + Nuts vs. Con): 0.82 (95% CI: 0.61–1.09) HR multivariate-adjusted A (Combined MedDiet vs. Con): 0.75 (95% CI: 0.58–0.96) HR multivariate-adjusted B (MedDiet + EVOO vs. Con): 0.60 (95% CI: 0.43–0.85) HR multivariate-adjusted B (MedDiet + Nuts vs. Con): 0.82 (95% CI: (0.61–1.10) HR multivariate-adjusted B (Combined MedDiet vs. Con): 0.70 (95% CI: 0.54–0.92)		
Effective translation of a type-2 diabetes primary prevention program into routine primary care: the PreDE cluster randomized clinical trial	Sanchez et al. (2018) [24]	Spain	Educational sessions to promote healthy lifestyle and provide information concerning diets and exercise. Continuous reinforcement through regular contact via telephone calls from nurses.	Control: 77 Intervention: 38	Unadjusted RR (intention to treat): 0.68 (95% CI: 0.47–0.99, *p* = 0.048) Unadjusted RR (per protocol): 0.69 (95% CI: 0.47–1.00) Adjusted RR (intention to treat): 0.68 (95% CI: 0.49–0.95) Adjusted RR (per protocol): 0.56 (95% CI: 0.39–0.81)		Relative risk of type 2 diabetes was reduced by a statistically significant 32% in the intervention group compared to the control.
Quality and quantity of protein intake influence incidence of type 2 diabetes mellitus in coronary heart disease patients: from the CORDIOPREV study	de la Cruz-Ares et al. (2021) [25]	Spain	Modifying eating behavior. Allocated diet (low-fat or Med diet).	106	HR unadjusted model: 0.6008 (95% CI: 0.4064–0.8883, *p* = 0.0096) HR multivariable model 1: 0.5981 (95% CI: 0.4043–0.8848, *p* = 0.0199) HR multivariable model 2: 0.6385 (95% CI: 0.4257–0.9578, *p* = 0.0024)		Adopting a diet with more plant-based proteins was linked to a statistically significant 36% lower risk of developing type 2 diabetes in the intervention group compared to the control.
Prevention of type 2 diabetes in adults with impaired glucose tolerance: the European Diabetes Prevention RCT in Newcastle upon Tyne, UK	Penn et al. (2009) [26]	UK	Individual and group activities by dietitian and physiotherapist. Information pack detailing facilities and opportunities for physical activity in Newcastle upon Tyne with a City Card and the opportunity to meet with a trainer at a local leisure center.	Control: 11 Intervention: 5	Incidence rate per 1000 person-years: 67.1 (95% CI: 34.2–117.5)	Incidence rate per 1000 person-years: 32.7 (95% CI: 10.7–74.6)	The overall incidence of diabetes was reduced by a nonsignificant 55% in the intervention group compared to the control.
					RR: 0.45 (95% CI: 0.2–1.2)		
Effect of a lifestyle intervention on weight change in south Asian individuals in the UK at high risk of type 2 diabetes: a family-cluster randomized controlled trial	Bhopal et al. (2014) [27]	UK	Families had 15 visits from a dietitian over 3 years. Promoting a calorie-deficit diet and physical activity was emphasized. Advice included information on shopping and cooking.	Control: 17 Intervention: 12	OR adjusted: 0.68 (95% CI: 0.27–1.67, *p* = 0.3705)		The intervention group showed a lower frequency of progression to diabetes compared to the control group, though this difference was not statistically significant.
A community based primary prevention program for type 2 diabetes integrating identification and lifestyle intervention for prevention: the Let’s Prevent Diabetes cluster randomized controlled trial	Davies et al. (2016) [28]	UK	Increase knowledge and promote realistic perceptions of PDM, as well as promoting healthy behavior and physical activity. Modifying eating behavior.	Control: 67 Intervention: 64	Incidence rate per 1000 person-years (intention to treat and per protocol): 63.16 (95% CI: 49.71–80.24)	Incidence rate per 1000 person-years (intention to treat): 57.60 (95% CI: 45.09–73.59) Incidence rate per 1000 person-years (per protocol): 53.04 (95% CI: 40.31–69.80)	In the intervention group, there was a 26% reduction in the risk of developing type 2 diabetes compared to the control group, although this reduction was not statistically significant.
					HR: 0.74 (95% CI: 0.48–1.14, *p* = 0.18)		
A pragmatic and scalable strategy using mobile technology to promote sustained lifestyle changes to prevent type 2 diabetes in India and the UK: a randomized controlled trial	Nanditha et al. (2020) [29]	UK	Personalized education and motivation about healthy diet and the benefits of enhanced physical activity. Regular short message service (SMS) messages about lifestyle 2–3 times weekly during the trial.	Control: 56 Intervention: 64	HR: 0.99 (95% CI: 0.69–1.43)		Personalized education and motivation on healthy diet and physical activity, supported by SMS messages 2–3 times a week, resulted in a nonsignificant reduction in diabetes progression in the intervention group over two years.
Lifestyle intervention with or without lay volunteers to prevent type 2 diabetes in people with impaired fasting glucose and/or nondiabetic hyperglycemia: a randomized clinical trial	Sampson et al. (2021) [30]	UK	Support maintenance of changes in physical activity and diet using patient-centered counseling techniques. Behavior changes and increased motivation with individually tailored goal settings.	Control: 39 Intervention group 1: 55 Intervention group 2: 62	OR unadjusted (INT vs. CON): 0.53 (95% CI: 0.34–0.84, *p* = 0.01) OR unadjusted (INT-DPM vs. CON): 0.60 (95% CI: 0.3–0.93, *p* = 0.02) OR unadjusted (Combined INT vs. CON): 0.57 (95% CI: 0.38–0.85, *p* = 0.01) OR adjusted (INT vs. CON): 0.54 (95% CI: 0.34–0.85, *p* = 0.01) OR adjusted (INT-DPM vs. CON): 0.60 (95% CI: 0.38–0.94, *p* = 0.02) OR adjusted (Combined INT vs. CON): 0.57 (95% CI: 0.38–0.85, *p* = 0.01) OR adjusted (INT vs. CON): 0.54 (95% CI: 0.34–0.85, *p* = 0.01) OR adjusted (INT-DPM vs. CON): 0.61 (95% CI: 0.39–0.96, *p* = 0.03) OR adjusted (Combined INT vs. CON): 0.57 (95% CI: 0.38–0.87, *p* = 0.01)	OR unadjusted (INT-DPM vs. INT): 1.11 (95% CI: 0.7–1.65, *p* = 0.59) OR adjusted (INT-DPM vs. INT): 1.12 (95% CI: 0.75–1.65, *p* = 0.59) OR adjusted (INT-DPM vs. INT): 1.14 (95% CI: 0.77–1.70, *p* = 0.51)	Individuals classified in the high-risk intermediate glycemic category with impaired fasting glucose and/or nondiabetic hyperglycemia were 40% to 47% less likely to develop type 2 diabetes in the intervention groups compared to the control over an average period of 24 months.
					HR unadjusted (INT vs. CON): 0.53 (95% CI: 0.35–0.80, *p* = 0.003) HR unadjusted (INT-DPM vs. CON): 0.62 (95% CI: 0.41–0.92, *p* = 0.02) HR unadjusted (Combined INT vs. CON): 0.57 (95% CI: 0.40–0.82, *p* = 0.002) HR adjusted (INT vs. CON): 0.53 (95% CI: 0.35–0.81, *p* = 0.003) HR adjusted (INT-DPM vs. CON): 0.64 (95% CI: 0.43–0.97, *p* = 0.03) HR adjusted (Combined INT vs. CON): 0.58 (95% CI: 0.41–0.84, *p* = 0.004)	HR unadjusted (INT-DPM vs. INT): 1.09 (95% CI: 0.76–1.57, *p* = 0.63) HR adjusted (INT-DPM vs. INT): 1.13 (95% CI: 0.78–1.63, *p* = 0.51)	
Predictors of lifestyle intervention outcome and dropout: the SLIM study	Roumen et al. (2011) [31]	The Netherlands	Dietary recommendations and physical activity training. Personalized advice from researcher and/or dietitian.	N/A	RR: 0.53 (95% CI: 0.29–0.97, *p* = 0.04)		Lifestyle counseling was associated with a statistically significant diabetes risk reduction of 47% in the intervention group compared to the control.
A lifestyle intervention to reduce type 2 diabetes risk in Dutch primary care: 2.5-year results of a randomized controlled trial	Vermunt et al. (2012) [32]	The Netherlands	Consultations with the nurse practitioner and the general practitioner. Meetings with dieticians and physiotherapists to provide information on diet and exercise.	Control: 46 Intervention: 41	Cumulative incidence percentage (*p* = 0.14): 0.5 year: 3.9% 1.5 year:8.3% 2.5 year: 11.9%	Cumulative incidence percentage (*p* = 0.28): 0.5 year: 4.6% 1.5 year:8.1% 2.5 year: 10%	Cumulative diabetes incidence was not significantly different between the intervention and the usual care (control) group.

## Data Availability

No new data were created or analyzed in this study. Data sharing is not applicable to this article.

## Supplementary Materials

The following supporting information can be downloaded at https://www.mdpi.com/article/10.3390/nu17061053/s1. File S1: Customized search strategies utilized for every electronic database, alongside the respective count of studies retrieved from each database.

## References

[1] European Commission (2021). Knowledge for Policy: Health Promotion and Disease Prevention Knowledge Gateway. Diabetes preventions. https://knowledge4policy.ec.europa.eu/health-promotion-knowledge-gateway/diabetes-prevention_en.

[2] International Diabetes Federation (2021). IDF Diabetes Atlas.

[3] European Union (2020). Health at a Glance: Europe 2020: State of Health in the EU Cycle.

[4] Health and Environment Alliance (HEAL) (2014). Health Costs in the European Union—How Much Is Related to EDCS.

[5] Khan M.A.B., Hashim M.J., King J.K., Govender R.D., Mustafa H., Al Kaabi J. (2020). Epidemiology of Type 2 Diabetes—Global Burden of Disease and Forecasted Trends. J. Epidemiol. Glob. Health.

[6] World Health Organization (2016). Global Report on Diabetes.

[7] Skoglund G., Nilsson B.B., Olsen C.F., Bergland A., Hilde G. (2022). Facilitators and barriers for lifestyle change in people with prediabetes: A meta-synthesis of qualitative studies. BMC Public Health.

[8] Nikpour S., Mehrdad N., Sanjari M., Aalaa M., Heshmat R., Mafinejad M.K., Larijani B., Nomali M., Ghezeljeh T.N. (2022). Challenges of Type 2 Diabetes Mellitus Management From the Perspective of Patients: Conventional Content Analysis. Interact. J. Med. Res..

[9] Mahrouseh N., Lovas S., Njuguna D.W., Nellamkuzhi N.J., Soares Andrade C.A., Sackey W.E., Irawan A.S., Varga O. (2022). How the European Union legislations are tackling the burden of diabetes mellitus: A legal surveillance study. Front. Public Health.

[10] European Parliament European Parliament resolution of 23 November 2022 on Prevention, Management and Better Care of Diabetes in the EU on the Occasion of World Diabetes Day. https://www.europarl.europa.eu/doceo/document/TA-9-2022-0409_EN.html.

[11] International Diabetes Federation European Region Diabetes—The Policy Puzzle: Towards Benchmarking in the EU 25. https://health.ec.europa.eu/system/files/2016-11/policy_puzzle_benchmarking_eu25_0.pdf.

[12] Manios Y., Lambrinou C.P., Mavrogianni C., Cardon G., Lindström J., Iotova V., Tankova T., Rurik I., Stappen V.V., Kivelä J. (2020). Lifestyle Changes Observed among Adults Participating in a Family- and Community-Based Intervention for Diabetes Prevention in Europe: The 1st Year Results of the Feel4Diabetes-Study. Nutrients.

[13] OECD (2022). Healthy Eating and Active Lifestyles: Best Practices in Public Health.

[14] Tuomilehto J., Uusitupa M., Gregg E.W., Lindström J. (2023). Type 2 Diabetes Prevention Programs—From Proof-of-Concept Trials to National Intervention and Beyond. J. Clin. Med..

[15] Tuomilehto J., Lindström J., Eriksson J.G., Valle T.T., Hämäläinen H., Ilanne-Parikka P., Keinänen-Kiukaanniemi S., Laakso M., Louheranta A., Rastas M. (2001). Prevention of type 2 diabetes mellitus by changes in lifestyle among subjects with impaired glucose tolerance. N. Engl. J. Med..

[16] NHLBI Study Quality Assessment Tools. https://www.nhlbi.nih.gov/health-topics/study-quality-assessment-tools.

[17] Skaaby T., Jørgensen T., Linneberg A. (2018). A randomized general population study of the effects of repeated health checks on incident diabetes. Endocrine.

[18] Lindström J., Eriksson J.G., Valle T.T., Aunola S., Cepaitis Z., Hakumäki M., Hämäläinen H., Ilanne-Parikka P., Keinänen-Kiukaanniemi S., Laakso M. (2003). Prevention of diabetes mellitus in subjects with impaired glucose tolerance in the Finnish Diabetes Prevention Study: Results from a randomized clinical trial. J. Am. Soc. Nephrol..

[19] Lindström J., Ilanne-Parikka P., Peltonen M., Aunola S., Eriksson J.G., Hemiö K., Hämäläinen H., Härkönen P., Keinänen-Kiukaanniemi S., Laakso M. (2006). Sustained reduction in the incidence of type 2 diabetes by lifestyle intervention: Follow-up of the Finnish Diabetes Prevention Study. Lancet Lond. Engl..

[20] Lindström J., Peltonen M., Eriksson J.G., Ilanne-Parikka P., Aunola S., Keinänen-Kiukaanniemi S., Uusitupa M., Tuomilehto J., Finnish Diabetes Prevention Study (DPS) (2013). Improved lifestyle and decreased diabetes risk over 13 years: Long-term follow-up of the randomised Finnish Diabetes Prevention Study (DPS). Diabetologia.

[21] Salas-Salvadó J., Bulló M., Babio N., Martínez-González M.Á., Ibarrola-Jurado N., Basora J., Estruch R., Covas M.I., Corella D., Arós F. (2011). Reduction in the incidence of type 2 diabetes with the Mediterranean diet: Results of the PREDIMED-Reus nutrition intervention randomized trial. Diabetes Care.

[22] Costa B., Barrio F., Cabré J.J., Piñol J.L., Cos X., Solé C., Bolíbar B., Basora J., Castell C., Solà-Morales O. (2012). Delaying progression to type 2 diabetes among high-risk Spanish individuals is feasible in real-life primary healthcare settings using intensive lifestyle intervention. Diabetologia.

[23] Salas-Salvadó J., Bulló M., Estruch R., Ros E., Covas M.I., Ibarrola-Jurado N., Corella D., Arós F., Gómez-Gracia E., Ruiz-Gutiérrez V. (2014). Prevention of diabetes with Mediterranean diets: A subgroup analysis of a randomized trial. Ann. Intern. Med..

[24] Sanchez A., Silvestre C., Campo N., Grandes G., PredDE Group (2018). Effective translation of a type-2 diabetes primary prevention programme into routine primary care: The PreDE cluster randomised clinical trial. Diabetes Res. Clin. Pract..

[25] de la Cruz-Ares S., Gutiérrez-Mariscal F.M., Alcalá-Díaz J.F., Quintana-Navarro G.M., Podadera-Herreros A., Cardelo M.P., Torres-Peña J.D., Arenas-de Larriva A.P., Pérez-Martínez P., Delgado-Lista J. (2021). Quality and Quantity of Protein Intake Influence Incidence of Type 2 Diabetes Mellitus in Coronary Heart Disease Patients: From the CORDIOPREV Study. Nutrients.

[26] Penn L., White M., Oldroyd J., Walker M., Alberti K.G.M., Mathers J.C. (2009). Prevention of type 2 diabetes in adults with impaired glucose tolerance: The European Diabetes Prevention RCT in Newcastle upon Tyne, UK. BMC Public Health.

[27] Bhopal R.S., Douglas A., Wallia S., Forbes J.F., Lean M.E., Gill J.M., McKnight J.A., Sattar N., Sheikh A., Wild S.H. (2014). Effect of a lifestyle intervention on weight change in south Asian individuals in the UK at high risk of type 2 diabetes: A family-cluster randomised controlled trial. Lancet Diabetes Endocrinol..

[28] Davies M.J., Gray L.J., Troughton J., Gray A., Tuomilehto J., Farooqi A., Khunti K., Yates T., Let’s Prevent Diabetes Team (2016). A community based primary prevention programme for type 2 diabetes integrating identification and lifestyle intervention for prevention: The Let’s Prevent Diabetes cluster randomised controlled trial. Prev. Med..

[29] Nanditha A., Thomson H., Susairaj P., Srivanichakorn W., Oliver N., Godsland I.F., Majeed A., Darzi A., Satheesh K., Simon M. (2020). A pragmatic and scalable strategy using mobile technology to promote sustained lifestyle changes to prevent type 2 diabetes in India and the UK: A randomised controlled trial. Diabetologia.

[30] Sampson M., Clark A., Bachmann M., Garner N., Irvine L., Howe A., Greaves C., Auckland S., Smith J., Turner J. (2021). Lifestyle Intervention with or Without Lay Volunteers to Prevent Type 2 Diabetes in People with Impaired Fasting Glucose and/or Nondiabetic Hyperglycemia: A Randomized Clinical Trial. JAMA Intern. Med..

[31] Roumen C., Feskens E.J.M., Corpeleijn E., Mensink M., Saris W.H.M., Blaak E.E. (2011). Predictors of lifestyle intervention outcome and dropout: The SLIM study. Eur. J. Clin. Nutr..

[32] Vermunt P.W.A., Milder I.E.J., Wielaard F., de Vries J.H., Baan C.A., van Oers J.A., Westert G.P. (2012). A lifestyle intervention to reduce Type 2 diabetes risk in Dutch primary care: 2.5-year results of a randomized controlled trial. Diabet. Med..

[33] McHugh M.L. (2012). Interrater reliability: The kappa statistic. Biochem. Medica.

[34] World Health Organization (1999). Department of Noncommunicable Disease Surveillance. Definition, Diagnosis, and Classification of Diabetes Mellitus. Part 1: Diagnosis and Classification of Diabetes Mellitus.

[35] World Health Organization (2011). Use of Glycated Haemoglobin (HbA1c) in the Diagnosis of Diabetes Mellitus.

[36] Gumber A., Gumber L. (2017). Improving prevention, monitoring and management of diabetes among ethnic minorities: Contextualizing the six G’s approach. BMC Res. Notes.

[37] Michels K.B. (2008). The promise and challenges of population strategies of prevention. Int. J. Epidemiol..

[38] Platt J.M., Keyes K.M., Galea S. (2017). Efficiency or equity? Simulating the impact of high-risk and population intervention strategies for the prevention of disease. SSM-Popul. Health.

[39] Goveia P., Cañon-Montañez W., Santos D.P., Lopes G.W., Ma R.C.W., Duncan B.B., Ziegelman P.K., Schmidt M.I. (2018). Lifestyle Intervention for the Prevention of Diabetes in Women With Previous Gestational Diabetes Mellitus: A Systematic Review and Meta-Analysis. Front. Endocrinol..

[40] Knowler W.C., Barrett-Connor E., Fowler S.E., Hamman R.F., Lachin J.M., Walker E.A., Nathan D.M., Diabetes Prevention Program Research Group (2002). Reduction in the Incidence of Type 2 Diabetes with Lifestyle Intervention or Metformin. N. Engl. J. Med..

[41] Karamanakos G., Costa-Pinel B., Gilis-Januszewska A., Velickiene D., Barrio-Torrell F., Cos-Claramunt X., Mestre-Miravet S., Piwońska-Solska B., Hubalewska-Dydejczyk A., Tuomilehto J. (2019). The effectiveness of a community-based, type 2 diabetes prevention programme on health-related quality of life. The DE-PLAN study. PLoS ONE.

[42] Shirvani T., Javadivala Z., Azimi S., Shaghaghi A., Fathifar Z., Devender Bhalla H.D.R., Abdekhoda M., Nadrian H. (2021). Community-based educational interventions for prevention of type II diabetes: A global systematic review and meta-analysis. Syst. Rev..

[43] Dineen T.E., Bean C., Jung M.E. (2022). Implementation of a diabetes prevention program within two community sites: A qualitative assessment. Implement. Sci. Commun..

[44] Aziz Z., Absetz P., Oldroyd J., Pronk N.P., Oldenburg B. (2015). A systematic review of real-world diabetes prevention programs: Learnings from the last 15 years. Implement. Sci..

[45] Teoh K.W., Ng C.M., Chong C.W., Bell J.S., Cheong W.L., Lee S.W.H. (2023). Knowledge, attitude, and practice toward pre-diabetes among the public, patients with pre-diabetes and healthcare professionals: A systematic review. BMJ Open Diabetes Res. Care.

[46] de Jong M., Jansen N., van Middelkoop M. (2023). A systematic review of patient barriers and facilitators for implementing lifestyle interventions targeting weight loss in primary care. Obes. Rev. Off. J. Int. Assoc. Study Obes..

[47] Aziz Z., Riddell M.A., Absetz P., Brand M., Oldenburg B., Australasian Peers for Progress Diabetes Project Investigators (2018). Peer support to improve diabetes care: An implementation evaluation of the Australasian Peers for Progress Diabetes Program. BMC Public Health.

[48] Iqbal N. (2007). The burden of type 2 diabetes: Strategies to prevent or delay onset. Vasc. Health Risk Manag..

[49] Prevention or Delay of Type 2 Diabetes: Standards of Medical Care in Diabetes—2021|Diabetes Care|American Diabetes Association. https://diabetesjournals.org/care/article/44/Supplement_1/S34/30895/3-Prevention-or-Delay-of-Type-2-Diabetes-Standards.

[50] Beck J., Greenwood D.A., Blanton L., Bollinger S.T., Butcher M.K., Condon J.E., Cypress M., Faulkner P., Fischl A.H., Francis T. (2017). 2017 National Standards for Diabetes Self-Management Education and Support. Diabetes Educ..

[51] ElSayed N.A., Aleppo G., Aroda V.R., Bannuru R.R., Brown F.M., Bruemmer D., Collins B.S., Hilliard M.E., Isaacs D., Johnson E.L. (2022). 2. Classification and Diagnosis of Diabetes: Standards of Care in Diabetes—2023. Diabetes Care.

